# Prognostic Value of Exportin-7 and Its Association with *KRAS* Status and Autophagy Markers in Small Intestinal Adenocarcinoma

**DOI:** 10.3390/life16071193

**Published:** 2026-07-19

**Authors:** Jeong Won Kim, Kris Ylaya, Eun Joo Chung, Sun-Young Jun, Seung-Mo Hong, Joon-Yong Chung

**Affiliations:** 1 Department of Pathology, Kangnam Sacred Heart Hospital, Hallym University College of Medicine, Seoul 07441, Republic of Korea; jwkim@hallym.or.kr; 2 Laboratory of Pathology, Center for Cancer Research, National Cancer Institute, National Institutes of Health, Bethesda, MD 20892, USA; ylayakri@mail.nih.gov; 3 Radiation Oncology Branch, Center for Cancer Research, National Cancer Institute, National Institutes of Health, Bethesda, MD 20892, USA; chungeu@mail.nih.gov; 4 Department of Pathology, Incheon St. Mary’s Hospital, College of Medicine, The Catholic University of Korea, Seoul 21431, Republic of Korea; pathssun@gmail.com; 5 Department of Pathology, Asan Medical Center, University of Ulsan College of Medicine, Seoul 05505, Republic of Korea; smhong28@gmail.com; 6 Center for Cancer Research, National Cancer Institute, National Institutes of Health, Bethesda, MD 20892, USA

**Keywords:** XPO7, small intestinal adenocarcinoma, prognostic biomarker, autophagy, overall survival

## Abstract

Small intestinal adenocarcinoma (SIAC) is a rare malignancy with a rising incidence and poor prognosis. Although exportin-7 (XPO7) is typically characterized as a tumor suppressor, recent evidence suggests context-dependent oncogenic functions, yet its role in SIAC remains unexplored. This study evaluated the clinicopathological significance of XPO7 expression and its association with *KRAS* status, autophagy, and cancer stemness in 191 surgically resected primary SIAC cases using immunohistochemistry and automated digital quantification. XPO7 was significantly upregulated in SIAC compared to normal mucosa (*p* < 0.001). High XPO7 expression, observed in 27.2% of cases, correlated with higher histological grade (*p* = 0.023) and was identified as an independent predictor of poor overall survival (hazard ratio = 1.703; *p* = 0.008). Subgroup analysis revealed that high XPO7 levels were associated with significantly shorter median overall survival specifically in cases featuring lymphovascular invasion, nodal metastasis, and *KRAS* mutations. Furthermore, XPO7 expression demonstrated a significant positive correlation with autophagy markers (LC3B and p62) but not with stemness markers (Sox2, Oct4, and Nanog). In conclusion, XPO7 serves as a significant independent prognostic biomarker for SIAC, and its correlation with autophagy markers in aggressive subgroups highlights its potential as a promising therapeutic target.

## 1. Introduction

The small intestine comprises approximately 75% of the total length of the gastrointestinal tract and nearly 90% of its mucosal surface area, yet it remains an uncommon site of malignancy, accounting for only ~3% of all gastrointestinal cancers [1]. Within this rare group, small intestinal adenocarcinoma (SIAC) is a predominant yet poorly understood histology, representing approximately 30–40% of primary small bowel cancers [2,3]. In contrast to the stable or declining incidence observed in many other gastrointestinal cancers including colorectal cancer [4,5], the incidence of SIAC has shown a gradual but consistent increase. Analysis of the Surveillance, Epidemiology, and End Results (SEER) Program database (2000–2019) reported an annual rise of approximately 0.3%, with disproportionately higher increases among men and individuals aged over 70 years [6]. Despite this trend, survival outcomes have remained largely unchanged, with 5-year overall survival (OS) rates showing minimal improvement over the past two decades [6,7]. This stagnation is largely attributable to delayed diagnosis, as SIAC is frequently clinically silent and anatomically inaccessible. Indeed, historical data indicate that SIAC is identified in only ~0.3% of autopsies [8] and up to 90% of patients present with advanced-stage disease (stage III or IV) at diagnosis [9,10].

Recent molecular profiling studies have further demonstrated that SIAC is not merely an extension of colorectal cancer biology but rather a distinct genomic entity. Compared with colorectal cancer, SIAC exhibits a markedly lower frequency of *APC* mutations, alongside a higher prevalence of *CDKN2A* alterations and activating mutations in *ERBB2*. In addition, SIAC shows enrichment for high tumor mutational burden (TMB) and microsatellite instability–high (MSI-H) phenotypes [11,12]. These findings challenge the conventional practice of extrapolating therapeutic strategies from colorectal cancer and underscore the need for SIAC-specific molecular characterization and biomarker-driven approaches.

Nucleocytoplasmic transport, primarily mediated by karyopherin-*β* receptors, regulates protein and RNA distribution. Although exportin-1 (XPO1) has been the most extensively characterized nuclear exporter in cancer biology [13], emerging evidence indicates that exportin-7 (XPO7), a member of the same superfamily, plays an increasingly recognized role in human [14,15]. While initially characterized as a tumor suppressor in developmental pathways [16,17], recent functional genomic and molecular profiling screens in prostate and breast cancers have revealed its paradoxical role as an oncogenic driver associated with poor clinical outcomes [18,19]. This functional divergence is largely attributed to its capacity to orchestrate nucleocytoplasmic trafficking of critical signaling molecules, which subsequently modulates oncogenic signaling cascades and cell-autonomous stress-adaptive mechanisms [20]. In particular, growing literature underscores that altered nuclear transport kinetics directly influence autophagic flux by controlling the dynamic shuttling and spatial partitioning of master transcription factors, such as transcription factor EB (TFEB), and critical nutrient sensors under metabolic stress [21]. Despite these evolving insights, the specific expression profile and clinical significance of XPO7 in SIAC remain completely unexplored. Given that SIAC is characterized by a distinct genomic landscape and a high propensity for advanced-stage presentation [9,10,11,12], identifying novel transport-dependent biomarkers linked to metabolic survival and transcriptional reprogramming strategies is imperative to improve biological risk stratification in this rare malignancy [22].

In the present study, we aimed to evaluate the protein expression of XPO7 in a large multi-institutional cohort of SIAC and analyze its correlation with clinicopathological parameters to determine its potential as an independent prognostic factor. We hypothesized that XPO7 expression correlates with adverse clinicopathological parameters and serves as an independent prognostic factor, particularly within the context of *KRAS* mutational status. Furthermore, to explore potential stress-adaptive networks at the tissue level, we investigated the molecular associations between XPO7 and established markers of autophagic flux, specifically LC3B and p62. These specific markers were strategically prioritized based on established clinical evidence demonstrating their robust prognostic relevance and functional utility in predicting survival outcomes within *KRAS*-mutated gastrointestinal malignancy [23]. This integrated baseline profiling aims to provide an exploratory, tissue-level framework for transport-dependent survival strategies in SIAC progression.

## 2. Materials and Methods

### 2.1. Study Cohort and Tissue Specimens

This study utilized a previously described cohort of 197 primary SIACs, which were surgically resected and collected from 22 South Korean institutions via the Korean Small Intestinal Cancer Study Group [24]. Inclusion was restricted to carcinomas arising from the mucosa of the duodenum, jejunum, or ileum. Tissue microarrays (TMAs) were constructed using all 197 surgically resected cases to ensure high-throughput and uniform immunohistochemical analysis. Among the initial pool, 191 cases (97.0%) provided sufficient tumor cellularity and high-quality tissue cores for automated digital analysis of XPO7 expression. To further investigate the molecular landscape, potential associations between XPO7 and other oncogenic markers were analyzed. These included *KRAS* mutational status (*n* = 186), autophagy markers (LC3B and p62; *n* = 171), and cancer stem cell (CSC) markers (Sox2, Oct4, and Nanog; *n* = 185), using data previously published from the same patient cohort (Supplementary Figure S1). The study protocol was approved by the Institutional Review Board of Incheon St. Mary’s Hospital (No. OC26SIDI0093, Seoul, Republic of Korea) and conducted in compliance with the Declaration of Helsinki, with the requirement for informed consent being waived by the IRB due to the retrospective nature of this study.

### 2.2. Clinicopathological Data Evaluation

**Figure 1:**
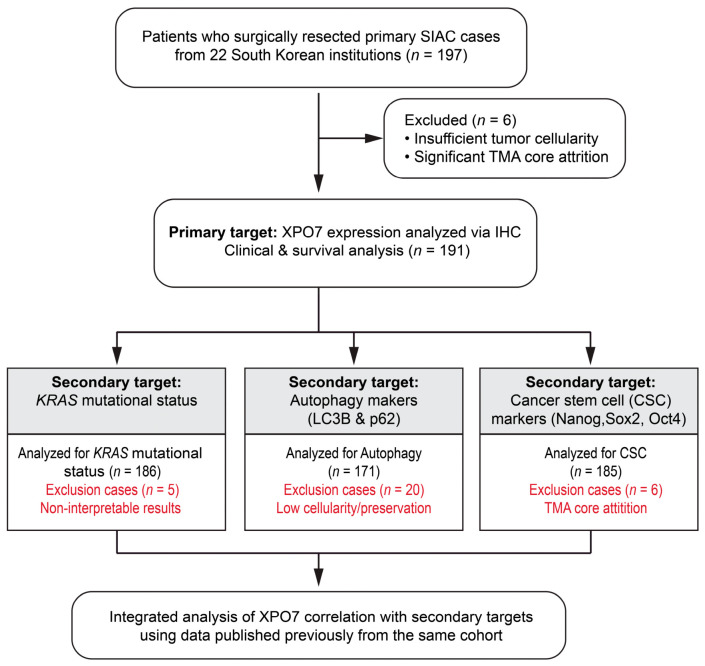
Study flowchart for patient selection and molecular analysis.

Baseline clinical and pathologic characteristics were retrieved from the previous study database [24]. Clinical parameters included patient demographics, tumor location, surgical records, and survival status. Tumors were staged according to the 8th edition of the American Joint Committee on Cancer (AJCC) cancer staging system [25]. We also reviewed predisposing conditions such as Lynch syndrome, Crohn’s disease, and familial adenomatous polyposis. Pathologic assessment involved tumor size and macroscopic growth patterns, categorized as polypoid, nodular, or infiltrative. Microscopic evaluation included histologic subtyping and grading (low- vs. high-grade) based on the 5th edition of the World Health Organization (WHO) classification [26], along with assessments of invasion depth, lymphovascular invasion (LVI), perineural invasion, and nodal status. Furthermore, a standardized CONSORT-style flowchart outlining the detailed screening workflow, predefined exclusion pathways for cases with insufficient tumor tissue density or incomplete clinical records, and the finalized data availability for each downstream statistical modality across the 22 collaborating institutions is systematically presented in Figure 1.

### 2.3. Immunohistochemistry

Immunohistochemical analysis was performed on previously validated TMAs [27], constructed from archived formalin-fixed, paraffin-embedded (FFPE) SIAC specimens. Triplicate 1.0 mm tumor cores and a single matched normal mucosal core per case were obtained using a manual tissue arrayer (Beecher Instruments, Sun Prairie, WI, USA). Sections were deparaffinized in xylene, rehydrated through graded alcohols, and subjected to heat-induced epitope retrieval in a Pascal pressure chamber (Pascal; Dako, Carpinteria, CA, USA) using Trilogy buffer (pH 9; Cell Marque/Millipore Sigma, Rocklin, CA, USA). Endogenous peroxidase activity was quenched with 3% hydrogen peroxide, followed by protein blocking to reduce nonspecific binding. Slides were incubated with a rabbit polyclonal anti-RanBP16/XPO7 antibody (Cat# NBP1-32350; Novus Biologicals, Centennial, CO, USA; 1:200 dilution) for 30 min at room temperature. Detection was carried out using the EnVision+ Rabbit-HRP (Dako) with 3,3-diaminobenzadine (Cell Marque) as the chromogen, sections were counterstained with hematoxylin. Negative controls including immunoglobulin G (IgG) and omission of the primary antibody were concurrently performed, and the TMA included appropriate positive control tissues.

### 2.4. Quantitative Analysis of Immunohistochemical Staining

Digitized images of the stained sections were captured using an Aperio AT2 scanner (Leica Biosystems, Vista, CA, USA) at 40× magnification, followed by automated quantification of XPO7 protein expression subsequently quantified via Visiopharm software v6.9.1 (Visiopharm, Hørsholm, Denmark) utilizing pre-defined scoring parameters. The analytical algorithm specifically evaluated brown cytoplasmic chromogenic intensity because preliminary histopathological screening revealed that XPO7 expression was overwhelmingly localized to the cytoplasmic compartment in SIAC cells, whereas nuclear staining was exceptionally sparse and lacked sufficient statistical variability for meaningful prognostic stratification. This processing pipeline generated a continuous distribution of positive proportions ranging from 0% to 100%.

### 2.5. KRAS Mutational Analysis

The *KRAS* mutation profile for this cohort was established in a prior study [28]. Briefly, genomic DNA was isolated from FFPE tissue sections using the QIAmp DNA Mini Kit (Qiagen, Valencia, CA, USA) following the manufacturer’s protocol. Target regions spanning codons 12 and 13 of *KRAS* exon 1 were amplified via polymerase chain reaction (PCR) using high-fidelity Taq polymerase. Following amplification, the resulting PCR products were purified with the QIAquick PCR Purification Kit (Qiagen). Subsequent cycle sequencing was performed using the BigDye Terminator Cycle Sequencing Kit v1.1 (Applied Biosystems, Foster City, CA, USA) and analyzed on an ABI Prism 310 Genetic Analyzer (software v3.7; Applied Biosystems).

### 2.6. Assessment of MSI

MSI status was characterized based on data previously reported [29], utilizing a single multiplex PCR assay. This panel evaluated five mononucleotide repeat markers: BAT25, BAT26, NR21, NR24, and NR27. The amplified fragments were separated by capillary electrophoresis and processed using the ABI Prism 310 Genetic Analyzer (Applied Biosystems). Tumors were classified according to the criteria established by the National Cancer Institute. MSI-H was defined as instability in two or more of the five markers. Tumors exhibiting instability in none or only one marker were classified as microsatellite stable (MSS), with cases previously categorized as low-frequency MSI (MSI-L) included in the MSS group for subsequent analyses.

### 2.7. Analysis of Correlation Between XPO7 and Autophagy or CSC Markers

To evaluate the biological landscape of XPO7 in SIAC, we analyzed its relationship with autophagy-related proteins (LC3B and p62) and CSC markers (Sox2, Nanog, and Oct4). All marker expressions were quantified as continuous variables using digital image analysis to ensure objective and reproducible measurement. The expression data for autophagy markers and CSC markers were obtained from our previously published studies using the same patient cohort [27,30]. For the correlation analysis, we utilized Spearman’s pairwise correlation coefficient (*ρ*) to assess the strength and direction of the association between the continuous expression levels of XPO7 and these previously established markers.

### 2.8. Statistical Analysis

All statistical computations were executed using IBM SPSS Statistics (version 29.0; IBM Corp., Armonk, NY, USA). Continuous variables were compared using the paired or unpaired Student’s *t*-test, while associations between categorical clinicopathological features were evaluated using the χ2 test or Fisher’s exact test, as appropriate. To establish optimal stratification, XPO7 expression was dichotomized into high and low groups based on a cut-off value of 22.4%, which was determined using maximally selected rank statistics (Web-R platform; http://web-r.org, accessed on 1 June 2026). Survival outcomes were estimated via the Kaplan–Meier method and compared via the log-rank test. In the OS model, patient death from any cause was defined as an event, and surviving patients were censored at the date of their last follow-up. To identify independent prognostic indicators, univariable and multivariable analyses were conducted using Cox proportional hazards regression models. For the multivariable model, a strict two-step procedure was utilized. First, all clinicopathological variables that demonstrated clinical significance or achieved the standard statistical significance threshold of *p* < 0.05 in the univariable screening were considered candidates for inclusion, thereby excluding covariates that only approached marginal significance. Second, a backward stepwise elimination procedure was applied to finalize the model, during which clinically established confounding factors, including tumor stage and nodal status, were forced into the model to ensure robust clinical adjustment. To ensure the mathematical validity of our regression models, the proportional hazards assumption was formally verified using Schoenfeld residual plots and statistical tests, confirming that the assumption was not violated for any of the adjusted covariates (*p* > 0.05). Additionally, multicollinearity among the clinicopathological covariates was evaluated by calculating the variance inflation factor (VIF). All tested variables in the final multivariable models demonstrated a VIF well below 2.0, indicating negligible collinearity. All tests were two-sided, and a *p* value of less than 0.05 was maintained as the threshold for statistical significance.

## 3. Results

### 3.1. Clinicopathological Characteristics

The clinicopathological features of the 191 SIAC patients are summarized in Table 1. The median age was 60 years (range, 23–86 years), with a male predominance (120/191, 62.8%). The primary tumor locations were the duodenum (104/191, 54.5%), jejunum (57/191, 29.8%), and ileum (30/191, 15.7%). The majority of tumors were diagnosed at an advanced stage, with 90.1% (172/191) of cases classified as pT3 or pT4. Nodal metastasis was identified in 51.4% (89/173), and LVI and perineural invasion were observed in 51.8% (99/191) and 33.0% (63/191) of patients, respectively. MSI-H was present in 24.1% (46/191) of the patients, and *KRAS* mutations were identified in 32.3% (60/186). Regarding treatment, 38.5% (72/187) of the patients received adjuvant chemotherapy. The median follow-up duration for the entire cohort was 28.4 months (range, 0–168.4 months), during which 130 patients (68.1%) died.

### 3.2. XPO7 Expression and Its Relation to KRAS Status

**Figure 2:**
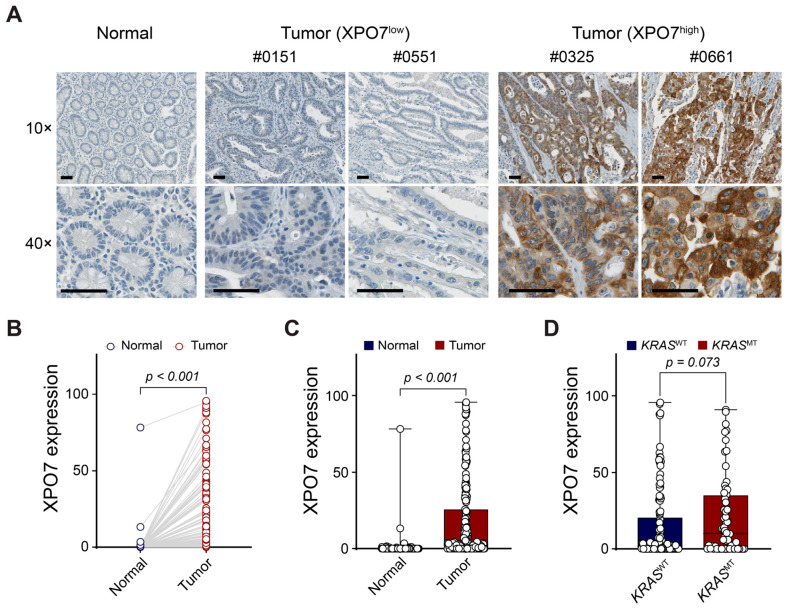
XPO7 expression in human small intestine adenocarcinoma (SIAC). (**A**) Representative immunohistochemical staining of XPO7 in normal intestinal mucosa and SIAC tissues, with tumors stratified into XPO7^low^ and XPO7^high^ groups based on staining intensity and scale bars indicating 50 µm (top, 10×; bottom, 40×). (**B**) Paired analysis of XPO7 expression in matched normal (*n* = 138) and tumor (*n* = 191) tissues, with each line representing a matched pair and showing a significant difference calculated via the Wilcoxon signed-rank test (*p* < 0.001). (**C**) Distribution of XPO7 expression across the full cohort, demonstrating significantly elevated levels in tumor tissues compared to normal controls calculated via the Mann–Whitney U test (*p* < 0.001). (**D**) Association between XPO7 expression and *KRAS* mutational status, showing a trend toward increased XPO7 levels in *KRAS*^MT^ (*n* = 60) versus *KRAS*^WT^ (*n* = 126) tumors (*p* = 0.073). Each circle represents an individual data point.

XPO7 expression was predominantly localized to the cytoplasm of tumor cells (Figure 2A and Figure S1). High XPO7 (XPO7^high^) expression was observed in 27.2% (52/191) of SIAC cases. To assess the differential expression of XPO7, we compared its levels between tumor tissues and matched non-adjacent normal intestinal mucosa from the exact same patients. Due to technical limitations inherent to TMA processing, including an insufficient histologic area of normal mucosal structures within certain archival donor cores for reliable evaluation or subsequent core detachment during sequential sectioning and immunohistochemical staining, paired normal mucosal counterparts were evaluable in 138 out of the 191 cases. In a paired analysis of 138 available cases, XPO7 expression was significantly elevated in tumor tissues compared to their normal counterparts (Figure 2B; *p* < 0.001). This upregulation was further supported by distributional analysis across the cohort, demonstrating a marked shift toward higher XPO7 expression in malignant samples (Figure 2C; *p* < 0.001). Correlation analysis with clinicopathological parameters revealed that XPO7^high^ was significantly associated with higher histological grade (*p* = 0.023), whereas no significant correlations were found with other clinicopathological parameters (Table 1).

Furthermore, we investigated the relationship between XPO7 expression and *KRAS* mutational status in 186 patients. XPO7 expression showed a marginal, non-significant trend toward higher levels in *KRAS*-mutant (*KRAS*^MT^) tumors compared with *KRAS* wild-type (*KRAS*^WT^) tumors (Figure 2D; *p* = 0.073). Similarly, the frequency of XPO7^high^ cases was higher in the *KRAS*^MT^ tumors (33.3% 20/60) than in the *KRAS*^WT^ tumors (23.8%, 30/126), though this difference did not reach statistical significance (*p* = 0.233; Table 1).

Stratified analyses based on *KRAS* genotype revealed distinct patterns. Within the *KRAS*^MT^ subgroup, XPO7^high^ was significantly associated with aggressive tumor features, including infiltrative growth (*p* = 0.046) and higher histological grade (*p* = 0.007) (Supplementary Table S1). In contrast, no such associations were observed in the *KRAS*^WT^ subgroup. These findings suggest that the biological impact of XPO7 may be context-dependent, with a more prominent role in promoting tumor aggressiveness in *KRAS*^MT^ SIAC.

### 3.3. Prognostic Significance of XPO7 Expression

**Figure 3:**
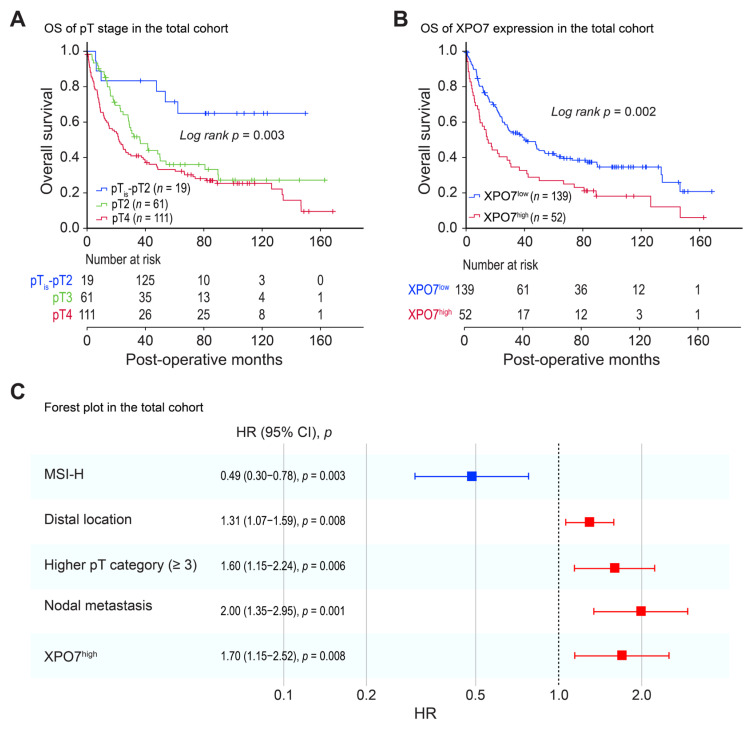
Prognostic value of XPO7 expression in SIAC. (**A**) Kaplan–Meier curves for overall survival (OS) stratified by pathologic tumor (pT) category show a significant decline in survival with advancing stage (*p* = 0.003). (**B**) Patients in the XPO7^high^ group exhibit significantly shorter OS compared to the XPO7^low^ group (*p* = 0.002). (**C**) Forest plot of multivariate Cox regression analysis, identifying XPO7^high^, distal tumor location, advanced pT category, and nodal metastasis as adverse prognostic factors and MSI-H as a favorable prognostic factor. The blue square represents the group with a favorable prognosis (HR < 1), and the red square represents the group with a poor prognosis (HR > 1).

Kaplan–Meier survival analysis revealed that advanced pathological T-category (pT) was strongly associated with adverse clinical outcomes. Patients with pT3 and pT4 disease exhibited significantly reduced OS rates (36.1% and 23.4%, respectively) compared with those in the early-T category group, who demonstrated an OS rate of 68.4% (*p* = 0.003; Figure 3A). Elevated XPO7 expression was also strongly correlated with poor prognosis. Patients with XPO7^high^ group had a substantially shorter median OS than those in the XPO7^low^ subset (14.4 months vs. 39.9 months, respectively; *p* = 0.002; Figure 3B, and further life-table analysis revealed that the 5-year OS rate for the entire cohort was 36.6%, which remained significantly lower in patients with high XPO7 expression (24.1%) compared to those with low expression (41.2%), further underscoring the long-term adverse prognostic impact of XPO7 upregulation in SIAC.

Given that XPO7 expression impacted outcome in SIAC patient’s outcome, we performed Cox proportional hazards regression analyses to identify the XPO7 as an independent prognostic factor. In the univariate analysis, distal location [hazard ratio (HR) = 1.266; 95% confidence interval (CI), 1.062–1.510; *p* = 0.009), advanced pT category (HR = 1.627; 95% CI, 1.224–2.162; *p* = 0.001), nodal metastasis (HR = 2.094; 95% CI, 1.433–3.059; *p* < 0.001), and XPO7^high^ status (HR = 1.751; 95% CI, 1.216–2.519; *p* = 0.003) were significant predictors of poorer OS. In contrast, MSI-H status (HR = 0.616; 95% CI, 0.400–0.948; *p* = 0.028) was significantly associated with improved OS (Table 2). After adjusting for potential confounders in multivariate model, XPO7^high^ status (HR = 1.703; 95% CI, 1.150–2.523; *p* = 0.008) remained a significant independent predictor of poor OS. Distal location (HR = 1.307; 95% CI, 1.072–1.593; *p* = 0.008), advanced pT category (HR = 1.604; 95% CI, 1.147–2.244; *p* = 0.006) and nodal metastasis (HR = 1.995; 95% CI, 1.347–2.954; *p* = 0.001) also remained independent adverse prognostic factors, whereas MSI-H status (HR = 0.485; 95% CI, 0.302–0.780; *p* = 0.003) retained its favorable prognostic significance (Table 2 and Figure 3C). These results indicate that XPO7 expression provides additional prognostic value beyond conventional pathological T and N staging.

**Figure 4:**
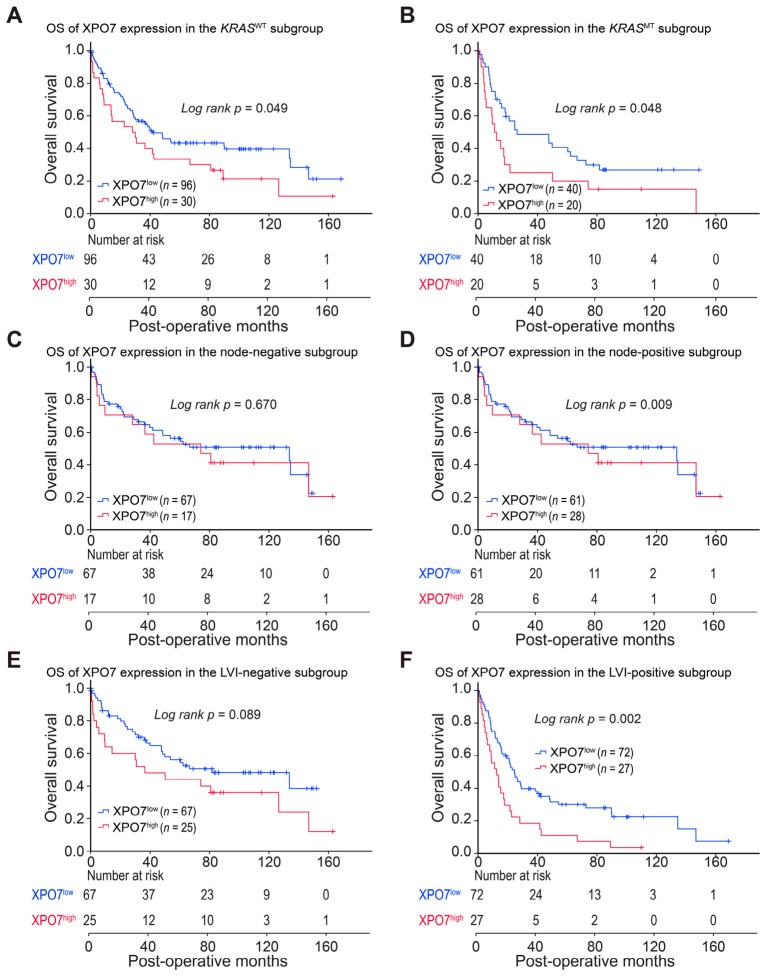
Subgroup analysis of the prognostic impact of XPO7 expression in SIAC. Kaplan–Meier curves of OS stratified by XPO7 expression across molecular and clinicopathological subgroups. Elevated XPO7 expression is associated with inferior OS in (**A**) *KRAS*^WT^ (*p* = 0.049) and (**B**) *KRAS*^MT^ (*p* = 0.048) cohorts, as well as in (**D**) node-positive (*p* = 0.009) and (**F**) lymphovascular invasion (LVI)-present disease (*p* = 0.002). No significant prognostic association was observed in (**C**) node-negative (*p* = 0.670), or (**E**) LVI-absent (*p* = 0.089).

Subgroup analyses further supported the prognostic relevance of XPO7. In both *KRAS*^WT^ and *KRAS*^MT^ cohorts, XPO7^high^ cohort was associated with significantly worse survival compared to XPO7^low^ (median OS: 28.1 vs. 41.7 months and 11.1 vs. 26.3 months, respectively; *p* = 0.049 and *p* = 0.048; Figure 4A,B). Similarly, among patients with nodal involvement or LVI, XPO7^high^ cases demonstrated significantly shorter OS than XPO7^low^ cases (node-positive: 13.7 vs. 26.6 months, *p* = 0.009; LVI-positive: 12.5 vs. 24.5 months, *p* = 0.002). In contrast, no significant survival differences were observed according to XPO7 expression in patients without nodal metastasis or LVI (Figure 4D–F). Collectively, these findings indicate that XPO7 expression serves as a robust prognostic indicator, particularly for stratifying high-risk patients within clinically aggressive subsets. However, given the limited sample sizes within these stratified cohorts, such as the *KRAS*^MT^ subgroup containing only 60 cases, and the borderline significance values achieved between 0.048 and 0.049, these subgroup findings must be interpreted with caution as exploratory observations rather than hypothesis-confirming evidence. The consistent association between XPO7 overexpression and reduced survival across both *KRAS*^MT^ and *KRAS*^WT^ populations highlights its potential utility as a broadly applicable biomarker for risk assessment.

### 3.4. Association of XPO7 with Autophagy and Stemness Markers

**Figure 5:**
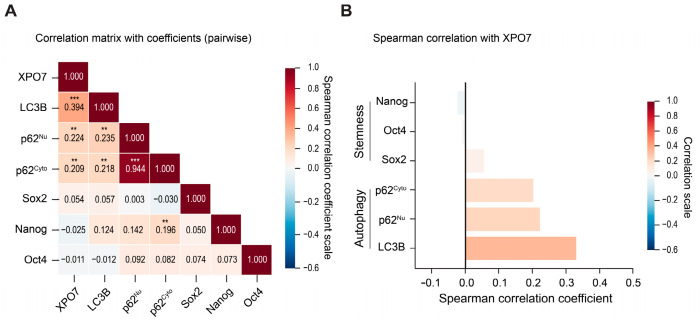
Correlation of XPO7 with autophagy (LC3B, p62) and CSC (Nanog, Sox2, OCT4) markers in SIAC. (**A**) Heatmap of Spearman’s rank correlation showing positive correlations with autophagy markers but no significant associations with CSC markers. (**B**) Spearman’s rank correlation analysis with bar colors indicating the direction and magnitude of correlations (red, positive; blue, negative) and significant positive correlations observed with autophagy markers. ** *p* < 0.05, *** *p* < 0.001.

To further elucidate the biological role of XPO7 in the context of SIAC, we analyzed its expression in relation to key markers of autophagy and cancer stemness (Figure 5). XPO7 expression showed a significant positive correlation with autophagy-related markers, whereas no meaningful association was observed with stemness-associated factors. Specifically, XPO7 expression was moderately correlated with LC3B (Spearman’s *ρ* = 0.394, *p* < 0.001), and weaker but still significant correlations were identified with both nuclear and cytoplasmic p62 (p62^nu^ and p62c^yto^; Spearman’s *ρ* = 0.224 and 0.209, respectively; *p* < 0.050; Figure 5A). These relationships are further illustrated in the bar plots, which demonstrate a consistently positive correlation between XPO7 and autophagy-related markers (Figure 5B). To provide a more detailed assessment of these relationships, we performed pairwise correlation analyses visualized in a scatter plot matrix (Supplementary Figure S2). The distribution and density of the data points reveal a consistent linear association between XPO7 and autophagy-related proteins, particularly LC3B. In contrast, XPO7 expression did not exhibit any statistically significant correlation with CSC markers, including Nanog, Sox2, and OCT4, with correlation coefficients remaining near zero. These findings suggest that XPO7 is more closely associated with autophagic activity in SIAC cells, rather than with the regulation of stem-like properties.

## 4. Discussion

The present study identifies XPO7 as a potential independent prognostic biomarker in SIAC. Subgroup analysis reveals that XPO7 expression significantly correlates with aggressive tumor phenotypes and worsened clinical outcomes. Specifically, XPO7^high^ in *KRAS*^MT^ cases is associated with a shorter median survival time compared to the *KRAS*^WT^ group. A consistent statistical association is found between XPO7 levels and established autophagy markers, while no such relationship is observed with cellular stemness. XPO7 may serve as a molecular indicator of autophagic activity in the SIAC microenvironment. Although the current study provides an observational framework, the significant association between XPO7 and autophagy-related signatures offers a promising basis for future research into the metabolic characteristics of advanced SIAC.

The paradoxical role of XPO7 as either a tumor suppressor or an oncogenic driver has been a subject of recent debate. While earlier studies suggested a tumor-suppressive role via Hedgehog signaling or p21-dependent senescence [16,17], our data align with recent genomic profiles in prostate and breast cancers that redefine XPO7 as an oncogenic driver [18,19]. This discrepancy suggests that XPO7-mediated nucleocytoplasmic transport is highly context-dependent, a plasticity that is likely governed by tissue-specific variations in available intracellular cargo loads, the differential expression of unique molecular binding partners, or complex reciprocal interactions with active oncogenic signaling pathways. Importantly, the significant correlation between XPO7^high^ and poor histological differentiation points toward an active role for XPO7 in facilitating aggressive phenotypic transformations in SIAC. This oncogenic shift likely stems from a disruption of epithelial integrity, where XPO7 may promote the epithelial-to-mesenchymal transition (EMT) by orchestrating the nuclear exclusion of key tumor suppressors. Such a process would effectively drive a loss of cellular identity, enabling cells to bypass senescence barriers and adopt the dedifferentiated, invasive phenotypes observed in our study. Given the association with advanced histological grade and the transition toward more mesenchymal-like states, the precise mechanisms by which the XPO7-specific interactome dictates these processes remain to be elucidated. Identifying the specific molecular cargo transported by XPO7 will be essential to understanding how this exportin drives the clinical progression of SIAC.

Members of the karyopherin exportin family regulate nucleocytoplasmic transport, a fundamental cellular process increasingly implicated in cancer biology and therapeutic resistance across gastrointestinal malignancies [20,31]. Our observation of a significant tissue-level correlation between XPO7 expression and the autophagy markers LC3B and p62 aligns with an emerging paradigm wherein nucleocytoplasmic transport machinery actively orchestrates metabolic stress adaptation [20]. Although the specific cargo specificity of XPO7 in small intestinal adenocarcinoma requires further biochemical verification, classical karyopherin and exportin family members have been increasingly shown to dynamically dictate the nucleocytoplasmic partitioning of master autophagy regulators, such as the transcription factor TFEB [21]. Under conditions of metabolic stress or hypoxia frequently encountered in the aggressive tumor microenvironment of SIAC, cells often hijack specific exportin networks to clear nuclear tumor suppressors or to redistribute essential signaling complexes to the cytoplasm, thereby sustaining autophagic flux and facilitating tumor cell survival. Furthermore, recent functional datasets underscore that aberrant nuclear transport signaling directly drives transcriptional programs associated with intracellular protein recycling and metabolic reprogramming [22]. While our clinical data are primarily correlative, these established crosstalk mechanisms in cancer biology provide a plausible biological rationale for the synchronized upregulation of XPO7 and autophagy components observed in our cohort.

Dysregulation of autophagy-related markers has been described in gastrointestinal cancers, including SIAC [30], and is generally considered to reflect heterogeneous alterations in autophagic activity and degradation rather than uniform pathway activation [32]. Autophagy exerts a pleiotropic and context-dependent influence on tumorigenesis, oscillating between tumor suppression and survival-promoting adaptation under metabolic or therapeutic stress [33,34,35]. Even though clinical efforts to inhibit autophagy with agents like hydroxychloroquine have shown pharmacodynamic activity in various solid tumors, definitive efficacy remains elusive [36,37,38]. In this context, the prognostic significance of XPO7 in the *KRAS*^MT^ subgroup aligns with the growing focus on precision oncology for SIAC. Given that autophagy is frequently activated as a survival mechanism in *KRAS*-driven malignancies [39], our findings identify a novel association between XPO7 and an autophagy-related molecular signature in SIAC. Ultimately, these insights position XPO7 as a novel and pivotal component within the regulatory network governing tumor metabolic pathways, reinforcing the value of targeting transport-dependent mechanisms in this rare malignancy. Furthermore, the independent prognostic capacity of XPO7, despite its minimal correlation with conventional clinicopathological parameters, suggests that this biomarker captures a distinct biological axis that diverges from macro-morphological tumor staging systems. While traditional parameters focus primarily on physical tumor expansion, XPO7 appears to serve as a cell-autonomous indicator of metabolic reprogramming and stress adaptation, whereby transport-driven autophagic flux shields aggressive tumor cell subsets against microenvironmental or therapeutic exhaustion. This independent functional survival axis explains why XPO7 overexpression reliably dictates long-term survival outcomes regardless of standard anatomical metrics, showcasing its unique utility as a molecular surrogate for biological risk stratification in SIAC patients.

Regarding therapeutic potential, our data provide a compelling baseline rationale for studying XPO7 in SIAC. Although selective inhibitors of nuclear export, most notably the FDA-approved XPO1 inhibitor selinexor, a selective inhibitor of nuclear export (SINE) compounds, primarily target XPO1 [36] and are actively undergoing clinical trials in gastrointestinal malignancies, it is critical to note that selective small-molecule XPO7 inhibitors are currently unavailable and remain to be developed for clinical testing, which contextualizes our conceptual propositions within an early translational framework. Nevertheless, the prognostic significance of XPO7 upregulation specifically within the *KRAS*^MT^ subgroup aligns with the growing focus on precision oncology for SIAC. While *KRAS* inhibitors like sotorasib and adagrasib have shown promise in other solid tumors [37], their efficacy in SIAC is still being explored. Because *KRAS-driven* malignancies characteristically employ protective autophagy as a vital metabolic survival mechanism to sustain tumor progression and resist therapeutic stress, our specific finding of a significant positive correlation between XPO7 levels and the expression of LC3B and p62 within the aggressive *KRAS*^MT^ cohort provides a robust and direct link to these pathways. Consequently, rather than proposing immediate therapeutic targeting, our data suggest that XPO7 could conceptually serve as a companion diagnostic tool to identify a distinct subpopulation of high-risk patients who might rationally benefit from combined therapeutic approaches targeting both transport-dependent survival networks and downstream autophagic flux, particularly in advanced cases where effective systemic treatment options are profoundly limited.

Although this study provides meaningful insights through a substantial scale retrospective analysis of multi-institutional samples, several limitations merit consideration. First, while our findings are established on a cohort from 22 institutions, the retrospective nature of the study may involve inherent selection bias. Furthermore, as our discovery was based on an Asian population, subsequent validation in diverse global cohorts comprising different racial and ethnic groups is required to ensure the universal prognostic robustness of XPO7. Second, the use of 1.0 mm diameter TMA cores presents an inherent limitation in capturing the full spectrum of intratumoral and intracellular heterogeneity of XPO7 expression. Because original whole-tissue sections were unavailable for a comparative uniformity check due to the multi-institutional, archived nature of this retrospective cohort, this potential sampling bias cannot be entirely ruled out. However, to meticulously minimize this constraint, we utilized triplicate tumor cores per case sampled from distinct, morphologically representative regions—an approach widely validated to maximize representative indexing and achieve high concordance with whole-slide staining. Third, the optimal cut-off value for XPO7 expression was derived from our specific dataset, which may limit immediate generalizability. This underscores the imperative for standardized scoring systems and validated thresholds in future clinical implementation. Fourth, although we confirmed that XPO7 expression is completely independent of microsatellite instability status (*p* = 0.993) and retains its powerful prognostic value after multivariable adjustment for this key genomic feature, our current tissue microarray infrastructure did not encompass comprehensive next-generation sequencing to systematically evaluate whether XPO7 serves as a surrogate for other recurrent somatic mutations, such as *TP53*, *SMAD4*, or *APC* alterations. Consequently, while our findings strongly point toward a novel cell-autonomous survival axis, future integrated genomic and proteomic co-profiling investigations will be necessary to fully map and validate XPO7 expression within the broader molecular landscape of SIAC. Furthermore, owing to the retrospective nature of this multi-institutional registry, specific recurrence timelines and exact progression dates were unavailable or incomplete for a substantial proportion of patients, which precluded the formal analysis of disease-free or progression-free survival. Fifth, despite the statistically significant correlation identified between XPO7 and autophagy markers LC3B and p62, we explicitly acknowledge that these observed associations are strictly correlative and observational rather than mechanistic. Because nucleocytoplasmic transport systems are known to regulate the intracellular trafficking of autophagy-related transcription factors under metabolic stress, we leveraged these two gold-standard readouts to screen for potential tissue-level networks. Notably, aberrant expression of LC3 and p62 has been established as a promising prognostic indicator in gastrointestinal malignancies, particularly demonstrating a close functional interplay within *KRAS*-mutated molecular backgrounds [23]. However, due to the lack of commercially available and well-characterized SIAC cell lines, in vitro mechanistic validations could not be performed in the current study. Additionally, because of the relatively small sample sizes in the molecular and clinicopathological sub-analyses, all subgroup survival data are presented strictly within an exploratory framework to inspire future large-scale confirmatory investigations. Consequently, the precise molecular pathways and specific cargoes through which XPO7 modulates autophagic flux remain to be fully elucidated. Sixth, because our cohort gathered archival specimens from 22 participating centers over an extended period, patients did not receive a single uniform standardized regimen of postoperative adjuvant therapy. This clinical treatment heterogeneity and potential variance in systemic management strategies may have influenced overall survival outcomes, representing an inherent limitation when retrospectively studying rare malignancies. Finally, to bridge the gap between our current observational findings and clinical application, future studies utilizing emerging model systems, such as patient-derived organoids or xenografts will be indispensable. These independent cohorts and functional models will be required to determine the definitive functional relevance of XPO7 in SIAC biology. At present, no selective XPO7 inhibitors are commercially or clinically available, and our tissue-level descriptive data do not provide direct functional evidence to support the immediate therapeutic targeting of XPO7. Nevertheless, our findings may support a conceptual framework for future translational research. Such research will be required to evaluate whether long-term development of nuclear export interference or indirect autophagy inhibition strategies can be rationally prioritized in XPO7-defined patient subsets, particularly those with *KRAS* mutations who lack effective treatment options.

## 5. Conclusions

In summary, XPO7 expression is an independent prognostic biomarker in SIAC. This study links XPO7 to autophagic activity rather than cancer stemness, suggesting a functional role in tumor progression. Its enrichment in *KRAS* mutant tumors further supports its significance as a metabolic survival factor and precision biomarker. Collectively, these results implicate nucleocytoplasmic transport in SIAC pathogenesis and justify investigating transport dependent autophagy as a target in this rare malignancy.

## Figures and Tables

**Table 1:** Correlation between XPO7 expression and clinicopathologic features of SIAC patients.

Characteristics, No. (%)	Total	XPO7		*p*
		XPO7^low^ (*n* = 139)	XPO7^high^ (*n* = 52)	
Age				0.359
<60 years	93 (48.7)	71 (51.1)	22 (42.3)	
≥60 years	98 (51.3)	68 (48.9)	30 (57.7)	
Sex				0.954
Male	120 (62.8)	88 (63.3)	32 (61.5)	
Female	71 (37.2)	51 (36.7)	20 (38.5)	
Location				0.358
Proximal (duodenum)	104 (54.5)	79 (56.8)	25 (48.1)	
Distal (jejunum and ileum)	87 (45.5)	60 (43.2)	27 (51.9)	
Growth pattern (*n* = 183)				0.817
Polypoid	33 (18.0)	25 (18.9)	8 (15.7)	
Nodular	12 (6.6)	8 (6.1)	4 (7.8)	
Infiltrative	138 (75.4)	99 (75.0)	39 (76.5)	
Histological subtype				0.942
Tubular	174 (91.1)	126 (90.6)	48 (92.3)	
Non-tubular ^a^	17 (8.9)	13 (9.4)	4 (7.7)	
Grade				0.023
Low (well and moderately differentiated)	145 (75.9)	112 (80.6)	33 (63.5)	
High (poorly differentiated and undiffer- entiated)	46 (24.1)	27 (19.4)	19 (36.5)	
LVI				1.000
Absent	92 (48.2)	67 (48.2)	25 (48.1)	
Present	99 (51.8)	72 (51.8)	27 (51.9)	
Predisposing condition				0.616
Absent	171 (89.5)	123 (88.5)	48 (92.3)	
Present	20 (10.5)	16 (11.5)	4 (7.7)	
Pancreatic invasion				0.664
Absent	122 (63.9)	87 (62.6)	35 (67.3)	
Present	69 (36.1)	52 (37.4)	17 (32.7)	
Perineural invasion				0.641
Absent	128 (67.0)	95 (68.3)	33 (63.5)	
Present	63 (33.0)	44 (31.7)	19 (36.5)	
pT category				0.898
pT_is_-pT_2_	19 (9.9)	13 (9.4)	6 (11.5)	
pT_3_	61 (31.9)	45 (32.4)	16 (30.8)	
pT_4_	111 (58.1)	81 (58.3)	30 (57.7)	
pN category (*n* = 173)				0.131
pN_0_	84 (48.6)	67 (52.3)	17 (37.8)	
pN_1_ + pN_2_	89 (51.4)	61 (47.7)	28 (62.2)	
Stage group (*n* = 173)				0.108
0-I	15 (8.7)	10 (7.8)	5 (11.1)	
II	69 (39.9)	57 (44.5)	12 (26.7)	
III	89 (51.4)	61 (47.7)	28 (62.2)	
MSI status				0.993
MSS	145 (75.9)	105 (75.5)	40 (76.9)	
MSI-H	46 (24.1)	34 (24.5)	12 (23.1)	
*KRAS* genotype (*n* = 186)				0.233
*KRAS* ^WT^	126 (67.7)	96 (70.6)	30 (60.0)	
*KRAS* ^MT^	60 (32.3)	40 (29.4)	20 (40.0)	
Adjuvant chemotherapy (*n* = 187)				0.872
Absent	115 (61.5)	84 (62.2)	31 (59.6)	
Present	72 (38.5)	51 (37.8)	21 (40.4)	
Survival status				0.005
Alive	61 (31.9)	53 (38.1)	8 (15.4)	
Deceased	130 (68.1)	86 (61.9)	44 (84.6)	

^a^ The non-tubular types included mucinous carcinomas (*n* = 9), signet ring cell carcinomas (*n* = 4), and undifferentiated carcinoma (*n* = 4).

**Table 2:** Univariate and multivariate analyses of OS in SIAC patients.

Variables	Univariate		Multivariate	
	HR [95% CI]	*p*	HR [95% CI]	*p*
Older age (≥60 years)	1.278 [0.904–1.806]	0.164		
Female sex	1.098 [0.769–1.567]	0.607		
*KRAS* ^MT^	1.427 [0.991–2.053]	0.056		
MSI-H	0.616 [0.400–0.948]	0.028	0.485 [0.302–0.780]	0.003
Distal location	1.266 [1.062–1.510]	0.009	1.307 [1.072–1.593]	0.008
Non-tubular histological type	1.072 [0.997–1.152]	0.060		
High grade	1.083 [0.950–1.234]	0.231		
Higher pT category (≥pT_3_)	1.627 [1.224–2.162]	0.001	1.604 [1.147–2.244]	0.006
Nodal metastasis	2.094 [1.433–3.059]	<0.001	1.995 [1.347–2.954]	0.001
XPO7^high^	1.751 [1.216–2.519]	0.003	1.703 [1.150–2.523]	0.008

## Data Availability

The datasets generated and/or analyzed during the current study are available from the corresponding author on reasonable request.

## Supplementary Materials

The following supporting information can be downloaded at: https://www.mdpi.com/article/10.3390/life16071193/s1, Supplementary Table S1: Correlation between clinicopathological factors and XPO7 expression based on *KRAS* genotype in SIAC patients; Supplementary Figure S1: Representative immunohistochemical staining of XPO7 in SIAC; Supplementary Figure S2: Pairwise correlation matrix of XPO7 and autophagy-related proteins.

## Abbreviations

The following abbreviations are used in this manuscript:

AJCC	American Joint Committee on Cancer
CI	Confidence Interval
CSC	Cancer Stem Cell
EMT	Epithelial-to-Mesenchymal Transition
FDA	Food and Drug Administration
FFPE	Formalin-Fixed, Paraffin-Embedded
HR	Hazard Ratio
IgG	Immunoglobulin G
*KRAS*^MT^	*KRAS*-Mutant
*KRAS*^WT^	*KRAS* Wild-Type
LVI	Lymphovascular Invasion
MSI	Microsatellite Instability
MSI-H	Microsatellite Instability–High
MSI-L	Low-frequency Microsatellite Instability
MSS	Microsatellite Stable
OS	Overall Survival
PCR	Polymerase Chain Reaction
p62^nu^	Nuclear p62
p62^cyto^	Cytoplasmic p62
SEER	Surveillance, Epidemiology, and End Results
SINE	Selective Inhibitor of Nuclear Export
TFEB	Transcription Factor EB
TMA	Tissue Microarray
TMB	Tumor Mutational Burden
VIF	Variance Inflation Factor
WHO	World Health Organization
XPO1	Exportin-1
XPO7	Exportin-7
XPO7^high^	High XPO7 expression
XPO7^low^	Low XPO7 expression
