# Supplementary material for: Prognostic Value of Exportin-7 and Its Association with KRAS Status and Autophagy Markers in Small Intestinal Adenocarcinoma
Source: Life (Basel). 2026 Jul 19;16(7):1193. doi: 10.3390/life16071193 (PMC13412204; doi:10.3390/life16071193)
Supplement: Supplementary file 1 [file life-16-01193-s001.zip › life-4427898-supplementary.pdf]

Supplementary Table S1. Correlation between clinicopathological factors and XPO7 expression based on *KRAS* genotype in SIAC patients

Category, No. (%)	*KRAS* genotype					
	KRAS^{WT} (n = 126)			*KRAS*^{MT} (n = 60)		
	XPO7^{low}	XPO7^{high}	p	XPO7^{low}	XPO7^{high}	p
Age			0.397			1.000
< 60 years	49 (51.0)	12 (40.0)		21 (52.5)	10 (50.0)	
≥ 60 years	47 (49.0)	18 (60.0)		19 (47.5)	10 (50.0)	
Sex			0.812			0.925
Male	62 (64.6)	18 (60.0)		24 (60.0)	13 (65.0)	
Female	34 (35.4)	12 (40.0)		16 (40.0)	7 (35.0)	
Location			0.611			0.639
Proximal (duodenum)	52 (54.2)	14 (46.7)		26 (65.0)	11 (55.0)	
Distal (jejunum and ileum)	44 (45.8)	16 (53.3)		14 (35.0)	9 (45.0)	
Growth pattern[a]			0.655			0.046*
Polypoid	18 (20.0)	5 (17.2)		7 (17.9)	3 (15.0)	
Nodular	7 (7.8)	1 (3.5)		0	3 (15.0)	
Infiltrative	65 (72.2)	23 (79.3)		32 (82.1)	14 (70.0)	
Histological subtype			1.000			1.000
Tubular	85 (88.5)	27 (90.0)		38 (95.0)	19 (95.0)	
Non-tubular[b]	11 (11.5)	3 (10.0)		2 (5.0)	1 (5.0)	
Grade[c]			0.372			0.007*
Low	71 (74.0)	19 (63.3)		38 (95.0)	13 (65.0)	
High	25 (26.0)	11 (36.7)		2 (5.0)	7 (35.0)	
Lymphovascular invasion			0.543			0.411
Absent	43 (44.8)	16 (53.3)		22 (55.0)	8 (40.0)	
Present	53 (55.2)	14 (46.7)		18 (45.0)	12 (60.0)	
Predisposing condition			1.000			0.648
Absent	85 (88.5)	27 (90.0)		35 (87.5)	19 (95.0)	
Present	11 (11.5)	3 (10.0)		5 (12.5)	1 (5.0)	
Pancreatic invasion			0.481			1.000
Absent	65 (67.7)	23 (76.7)		20 (50.0)	10 (50.0)	
Present	31 (32.3)	7 (23.3)		20 (50.0)	10 (50.0)	
Perineural invasion			0.742			0.922
Absent	66 (68.8)	19 (63.3)		28 (70.0)	13 (65.0)	
Present	30 (31.2)	11 (36.7)		12 (30.0)	7 (35.0)	
pT category			0.752			0.975
pT_{is}-pT_2	9 (9.4)	4 (13.3)		4 (10.0)	2 (10.0)	
pT_3	36 (37.5)	12 (40.0)		9 (22.5)	4 (20.0)	
pT_4	51 (53.1)	14 (46.7)		27 (67.5)	14 (70.0)	
pN category[d]			0.448			0.345
pN_0	43 (49.4)	10 (38.5)		22 (56.4)	7 (38.9)	
pN_1+pN_2	44 (50.6)	16 (61.5)		17 (43.6)	11 (61.1)	
Stage group[d]			0.401			0.330
0-I	7 (8.0)	3 (11.5)		3 (7.7)	2 (11.1)	
II	36 (41.4)	7 (26.9)		19 (48.7)	5 (27.8)	
III	44 (50.6)	16 (61.5)		17 (43.6)	11 (61.1)	
MSI status			0.760			0.732
MSS	72 (75.0)	21 (70.0)		31 (77.5)	17 (85.0)	

MSI-H	24 (25.0)	9 (30.0)		9 (22.5)	3 (15.0)	
Adjuvant chemotherapy[e]			0.380			0.923
Absent	57 (61.3)	15 (50.0)		26 (65.0)	14 (70.0)	
Present	36 (38.7)	15 (50.0)		14 (35.0)	6 (30.0)	
Survival status			0.053			0.161
Alive	40 (41.7)	6 (20.0)		12 (30.0)	2 (10.0)	
Deceased	56 (58.3)	24 (80.0)		28 (70.0)	18 (90.0)	

KRAS^{WT}, *KRAS* Wild type; *KRAS*^{MT}, *KRAS* mutated type; XPO7^{low}, low XPO7 expression; high XPO7 expression.

*Statistically significant ($p < 0.05$)

[a]Calculated using only 178 cases with available information on growth pattern.

[b]The non-tubular types included mucinous carcinomas (n = 9), signet ring cell carcinomas (n = 4), and undifferentiated carcinoma (n = 4).

[c]Low grade includes well and moderately differentiated, while high grade is categorized as poorly differentiated and undifferentiated.

[d]Calculated with only 170 cases with available information on nodal metastasis and stage grouping.

[e]Calculated with only 183 cases with available information on nodal metastasis.

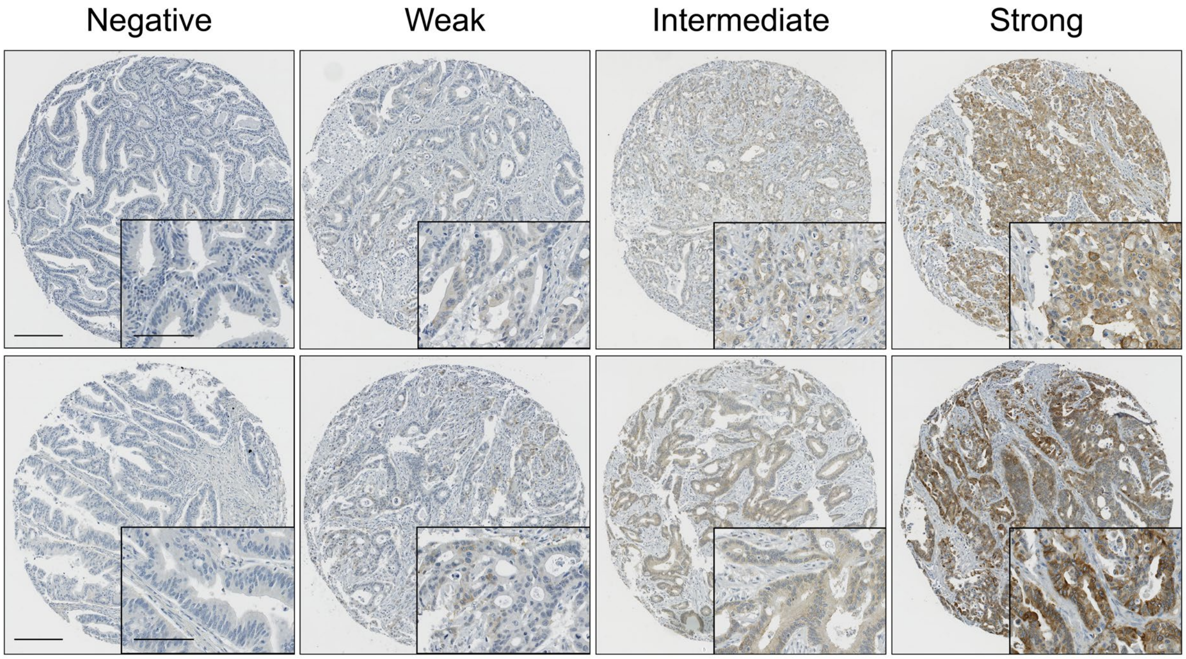

Supplementary Figure S1. Representative immunohistochemical staining of XPO7 in SIAC. Images display the semi-quantitative grading spectrum of XPO7 expression across tissue microarray cores, ranging from negative, weak, and intermediate to strong. Scale bars = 200 µm; insets = 100 µm.

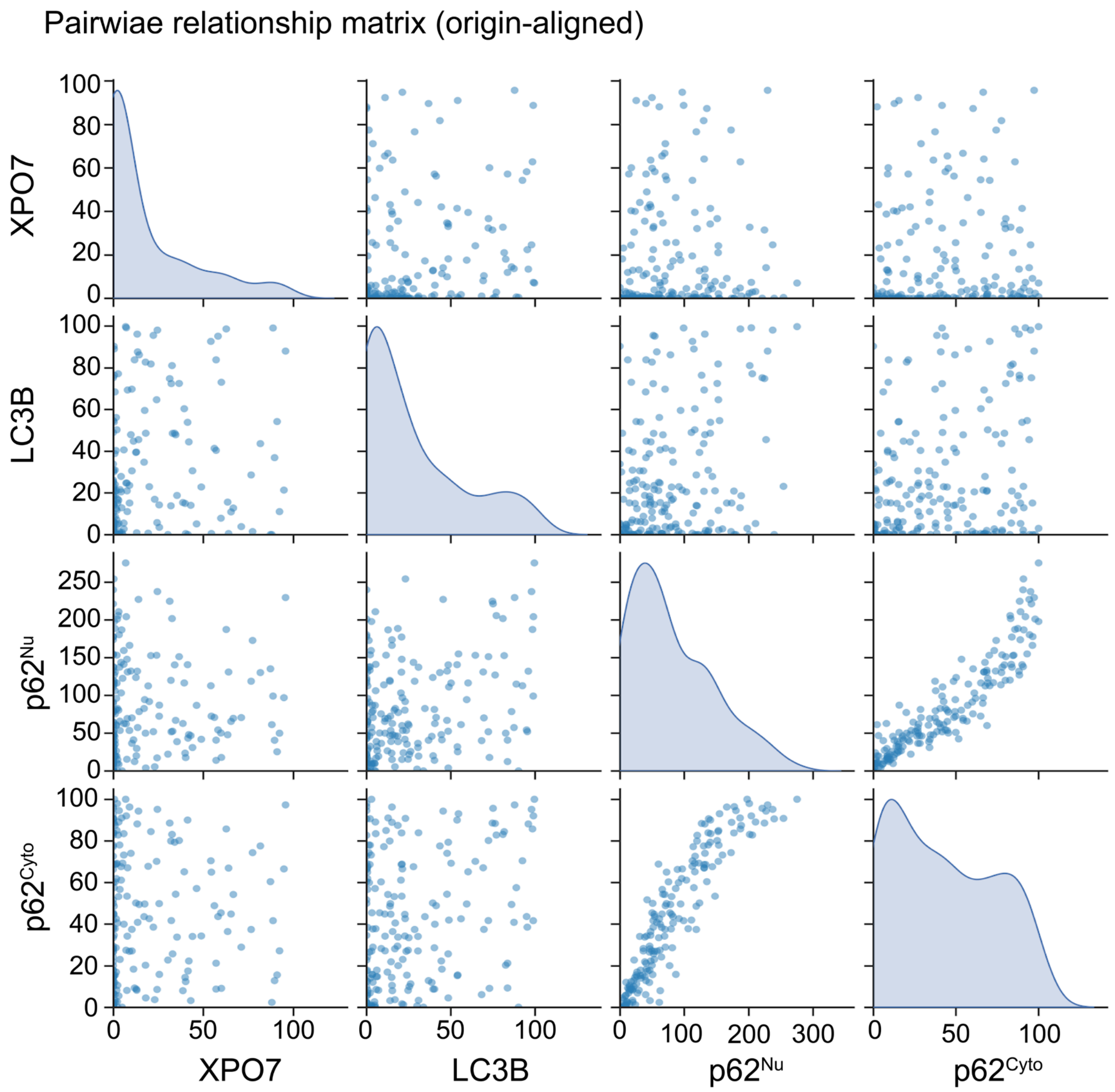

Supplementary Figure S2. Pairwise correlation matrix of XPO7 and autophagy-related proteins. The scatter matrix illustrates the pairwise relationship the continuous expression levels of XPO7, LC3B, nuclear p62 ($p62^{Nu}$), and cytoplasmic 62 ($p62^{Cyto}$). Diagonal plots represent the smoothed density distribution of each protein's expression levels within the cohort.
